# Spontaneous Double Closure of a Secondary Full-Thickness Macular Hole: A Case Report

**DOI:** 10.7759/cureus.101078

**Published:** 2026-01-08

**Authors:** Carolina Minelli Martines, Amanda Latuffe Soares Damião, Marina Gasparoni Teixeira Soares, Marcos R Franzosi, Eduardo Gallon

**Affiliations:** 1 Ophthalmology, Hospital das Clínicas, Faculty of Medicine, University of São Paulo (HCFMUSP), São Paulo, BRA

**Keywords:** full-thickness macular hole, idiopathic macular hole, pars plana vitrectomy (ppv), recurrent macular hole, reopen macular hole, spontaneously closed macular holes

## Abstract

Full-thickness macular holes (FTMHs) are defects of the central retina that cause clinically significant symptoms, including reduced visual acuity. Spontaneous closure may occur in a minority of cases, and pars plana vitrectomy (PPV) remains the gold-standard treatment. This report describes a patient who had previously undergone PPV and, during follow-up, was diagnosed with an FTMH at the Hospital das Clínicas, Faculty of Medicine, University of São Paulo (HCFMUSP), for whom surgical intervention was initially indicated. Prior to the scheduled surgery, the macular hole (MH) closed spontaneously. Several months later, the lesion recurred, and vitrectomy was again recommended. However, during preoperative follow-up, a second spontaneous closure was observed. To date, the patient has remained under clinical observation, and no surgical intervention has been required for this condition. This case highlights the dynamic behavior of secondary FTMHs and emphasizes the importance of longitudinal optical coherence tomography (OCT) follow-up in selected patients.

## Introduction

The macula is responsible for high-resolution central vision, and pathological involvement of this region can lead to significant visual impairment and reduced quality of life. Among macular disorders, full-thickness macular holes (FTMHs) represent a clinically relevant condition, characterized by a complete defect of the foveal retinal layers extending from the internal limiting membrane to the outer retina.

The current understanding of FTMH pathophysiology attributes its development to abnormal vitreomacular adhesion and traction, rather than a spontaneous degenerative process [1,2]. Tractional forces acting on the macula due to abnormal vitreomacular interface separation, which lead to the formation of intraretinal cysts that may progress to retinal breaks and ultimately result in a full-thickness defect [3]. FTMHs may be primary or secondary to identifiable factors, including ocular trauma, macular edema, inflammatory conditions, and prior vitreoretinal surgery [4].

Clinically, patients may present with decreased visual acuity, metamorphopsia, or central scotoma, although symptom severity varies according to hole size and stage. The condition occurs more frequently in women, with a 64% increased risk [5], and its incidence rises with advancing age, peaking between the sixth and seventh decades of life [6].

A clinical diagnosis can be made by slit-lamp biomicroscopy with fundus examination, in which a well-demarcated circular macular lesion is observed, often with yellowish deposits and frequently a surrounding cuff (halo) of subretinal fluid. The Watzke-Allen test may be used to support the clinical diagnosis during a noninvasive eye exam. 

Optical coherence tomography (OCT) has become the cornerstone of diagnosis and follow-up, allowing precise anatomical characterization of macular holes (MHs) and assessment of the vitreomacular interface [7]. The International Vitreomacular Traction Study (IVTS) Group classification provides a standardized approach to categorizing vitreomacular adhesion, traction, and MHs, thereby supporting consistent clinical decision-making and facilitating communication in both clinical practice and research settings [8].

While early-stage MHs may undergo spontaneous resolution, this phenomenon is uncommon once a full-thickness defect is established, with reported rates ranging from 3% to 10% [9]. Consequently, pars plana vitrectomy (PPV) remains the gold-standard treatment for FTMHs, achieving high anatomical closure rates [10]. Nonetheless, spontaneous closure of FTMHs has been reported, and the mechanisms and prognostic implications of this outcome remain a matter of debate.

In this report, we describe a patient with an FTMH, likely secondary to prior PPV, who demonstrated spontaneous closure of the lesion on two separate occasions during follow-up, without additional surgical intervention. This case highlights an unusual clinical course and contributes to the ongoing discussion regarding the natural history and management of FTMHs.

## Case presentation

**Figure 1:**
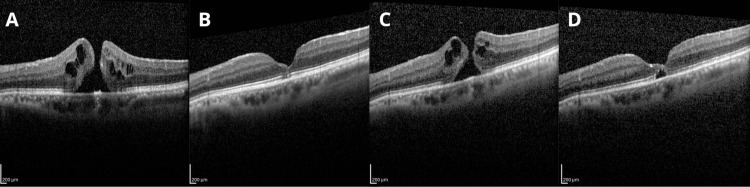
OCT images FTMH: full-thickness macular hole; MLD: minimum linear diameter; OCT: optical coherence tomography (A) OCT performed on day 1, showing the presence of a FTMH, MLD of 154 µm, basal diameter (BD) of 360 µm, hole height (HH) of 550 µm; (B) OCT performed at month 11, showing spontaneous closure of the FTMH; (C) OCT performed at month 15, showing a new FTMH, MLD of 104 µm, BD of 280 µm, HH 440 µm; (D) OCT performed at month 22, showing spontaneous closure of the FTMH

This is the case of a 66-year-old male patient with a medical history of diabetes mellitus for 10 years, with poor control. Ophthalmic history includes PPV for rhegmatogenous retinal detachment (RRD) followed by cataract surgery; both procedures were performed on the left eye (LE). At the initial evaluation, the patient presented to the Ophthalmology Department of the Hospital das Clínicas, Faculty of Medicine, University of São Paulo (HCFMUSP), with a one-month history of decreased visual acuity and metamorphopsia in the LE. Initial evaluation revealed reduced visual acuity in the LE, 20/40, on physical examination. Fundus examination and OCT demonstrated a Gass stage 2 FTMH (Figure 1A), classified as small (≤250 µm) according to the IVTS OCT-based classification, leading to the diagnosis of the MH. Consequently, PPV was indicated. During follow-up, the patient showed improvement in metamorphopsia, and a new OCT, performed after 11 months of initial presentation, demonstrated spontaneous closure of the MH (Figure 1B). The previously scheduled surgical intervention was therefore deemed unnecessary.

Fifteen months after the initial presentation, the patient returned with complaints of decreased visual acuity. On re-evaluation, the best-corrected visual acuity was 20/40 in the LE and 20/32 in the right eye. Fundus examination of the LE revealed an open MH associated with involvement of the temporal epiretinal membrane (ERM), in addition to a superotemporal laser scar. Recurrence of the FTMH at the same location, with associated temporal ERM involvement, was therefore diagnosed (Figure 1C). Surgical management was again indicated. However, at the 22nd month of follow-up, repeat OCT demonstrated a second spontaneous closure of the MH (Figure 1D), leading to cancellation of the planned vitrectomy. The patient was advised to return for follow-up in six months, given the possibility of reopening. After 26 months of the first presentation, the MH remained closed, and a strategy of close observation with semiannual follow-up was adopted.

## Discussion

Recent studies have substantially improved the understanding of MH formation and closure, particularly through advances in OCT-based evaluation of the vitreomacular interface [1-3,7]. Secondary FTMH after PPV for RRD is rare, with an incidence of approximately 1%, and typically occurs within the first months postoperatively [4]. A more recent case-control study evaluating risk factors for MH development after PPV for RRD found a higher risk among patients who developed postoperative cystoid macular edema (CME), supporting the idea that postoperative inflammation and edema-related foveal vulnerability can contribute to hole formation in addition to traction [11]. Most patients who undergo vitreoretinal surgery will not develop a secondary hole, but the risk is still meaningful, so follow-up should include symptom-guided OCT assessment. In the present case, patient-reported symptoms prompted evaluation on both occasions, and OCT was essential to confirm the diagnosis, document regression, and guide the timing of intervention versus observation.

Classification systems remain valuable for guiding prognosis and clinical management. MHs can be described using the clinical Gass staging system and an OCT-based anatomical classification. From a practical standpoint, OCT-based metrics are particularly helpful because they provide objective, reproducible measurements [7,8]. The IVTS Group subclassifies FTMHs by minimum linear diameter as small (≤250 µm), medium (>250 to 400 µm), and large (≥400 µm), and also considers whether vitreomacular traction (VMT) is present [8]. In the present report, the findings were most consistent with a small secondary MH, a profile that is more often compatible with spontaneous closure than larger defects [8,9].

Spontaneous closure of FTMH has been described in a minority of cases (approximately 3% to 15%) and often occurs within a few months after diagnosis, with potential for meaningful visual improvement [4,12,13]. This phenomenon is reported more frequently in smaller holes, especially those under 250 µm, and OCT features such as bridging tissue and suspended hyperreflective material have been linked to the likelihood of closure [9,14]. Several mechanisms have been proposed, including Müller cell-related tissue bridging that stabilizes the fovea, release of residual tractional forces at the fovea, and the formation with subsequent contraction of an ERM that may generate centripetal forces [4,7,9]. Sex, race, and other demographic characteristics have not been associated with differences in the frequency of spontaneous MH closure [14].

In secondary FTMH after PPV, persistent “classic” VMT is unlikely to be the only driver of foveal changes. Postoperative remodeling and tangential traction related to ERM or epiretinal proliferation may be more important contributors in some eyes [4,15]. Consistent with this, secondary FTMH has been reported to present more often with ERM and epiretinal proliferation than idiopathic FTMH, and a subretinal fluid cuff may also be seen on OCT, suggesting a mixed tractional and postoperative inflammatory environment [15]. In our patient, intermittent tractional disequilibrium related to postoperative membrane dynamics likely altered the balance of forces at the fovea, resulting in an unusual clinical course characterized by spontaneous closure, subsequent reopening, and eventual re-closure of the MH. Although this mechanism is plausible, a definitive causal relationship cannot be established in the absence of consistent longitudinal biomarker documentation.

Regarding management, most secondary post-vitrectomy FTMHs are treated surgically, typically with repeat PPV, with generally favorable outcomes [4,10]. A recent series reported a high single-surgery closure rate (93.9%) and a spontaneous closure rate of 5%, highlighting that observation can be appropriate in selected patients when the hole is small, vision is stable or improving, and OCT suggests a trend toward closure [15]. This case highlights that spontaneous MH closure does not eliminate the risk of subsequent reopening, underscoring the need for close OCT-based follow-up and prompt reconsideration of surgical management in the event of anatomical or functional deterioration [4,15].

## Conclusions

Secondary FTMHs after PPV for RRD are uncommon but can affect visual recovery and may follow an atypical course, significantly affecting patients' quality of life. Clinicians should consider the possibility of spontaneous closure, particularly when the hole is small, and OCT suggests favorable features, but maintain careful follow-up because reopening can occur. While repeat PPV remains the gold-standard treatment when intervention is indicated, cases with recurrent spontaneous closure support an individualized approach, using symptom-guided OCT monitoring and prompt reconsideration of surgery if anatomy or vision worsens.
